# Financial Stress and Outcomes after Acute Myocardial Infarction

**DOI:** 10.1371/journal.pone.0047420

**Published:** 2012-10-24

**Authors:** Sachin J. Shah, Harlan M. Krumholz, Kimberly J. Reid, Saif S. Rathore, Aditya Mandawat, John A. Spertus, Joseph S. Ross

**Affiliations:** 1 Department of Internal Medicine, Massachusetts General Hospital, Boston, Massachusetts, United States of America; 2 Robert Wood Johnson Foundation Clinical Scholars Program, Department of Medicine, Yale University School of Medicine, New Haven, Connecticut, United States of America; 3 Section of Health Policy and Administration, Department of Epidemiology and Public Health and Section of Cardiovascular Medicine, Department of Medicine, Yale University School of Medicine, New Haven, Connecticut, United States of America; 4 Department of Cardiology, Mid America Heart Institute of St. Luke’s Hospital, Kansas City, Missouri, United States of America; 5 Department of Internal Medicine, Brigham and Women’s Hospital, Boston, Massachusetts, United States of America; 6 Department of Cardiology, University of Missouri-Kansas City, Kansas City, Missouri, United States of America; 7 Section of General Internal Medicine, Department of Medicine, Yale University School of Medicine, New Haven, Connecticut, United States of America; 8 Center for Outcomes Research and Evaluation, Yale-New Haven Hospital, New Haven, Connecticut, United States of America; The University of Hong Kong, Hong Kong

## Abstract

**Background:**

Little is known about the association between financial stress and health care outcomes. Our objective was to examine the association between self-reported financial stress during initial hospitalization and long-term outcomes after acute myocardial infarction (AMI).

**Materials and Methods:**

We used Prospective Registry Evaluating Myocardial Infarction: Event and Recovery (PREMIER) data, an observational, multicenter US study of AMI patients discharged between January 2003 and June 2004. Primary outcomes were disease-specific and generic health status outcomes at 1 year (symptoms, function, and quality of life (QoL)), assessed by the Seattle Angina Questionnaire [SAQ] and Short Form [SF]-12. Secondary outcomes included 1-year rehospitalization and 4-year mortality. Hierarchical regression models accounted for patient socio-demographic, clinical, and quality of care characteristics, and access and barriers to care.

**Results:**

Among 2344 AMI patients, 1241 (52.9%) reported no financial stress, 735 (31.4%) reported low financial stress, and 368 (15.7%) reported high financial stress. When comparing individuals reporting low financial stress to no financial stress, there were no significant differences in post-AMI outcomes. In contrast, individuals reporting high financial stress were more likely to have worse physical health (SF-12 PCS mean difference −3.24, 95% Confidence Interval [CI]: −4.82, −1.66), mental health (SF-12 MCS mean difference: −2.44, 95% CI: −3.83, −1.05), disease-specific QoL (SAQ QoL mean difference: −6.99, 95% CI: −9.59, −4.40), and be experiencing angina (SAQ Angina Relative Risk = 1.66, 95%CI: 1.19, 2.32) at 1 year post-AMI. While 1-year readmission rates were increased (Hazard Ratio = 1.50; 95%CI: 1.20, 1.86), 4-year mortality was no different.

**Conclusions:**

High financial stress is common and an important risk factor for worse long-term outcomes post-AMI, independent of access and barriers to care.

## Introduction

Financial stress is the experience of financial anxiety, being in debt that cannot be paid off easily, and not being able to afford essential consumer items such as food and clothing. However, financial stress is an individualized experience dependent upon a person’s stress associated with expected financial loss, risk, or uncertainty [1]. In today’s American economy, burdened by an economic recession and high rates of unemployment, financial stress is common. Worsening matters is the prominent role that health care costs may play in creating financial stress, as 20% of Americans currently report medical debt [2] and health care bills substantially contribute to more than half of bankruptcies in the U.S. [3], [4].

Little is known about the association between financial stress and both health and health care outcomes, as previous research has not differentiated financial stress from access and other barriers to care [5], [6], [7]. Clearly, the association between both lacking health insurance and lower incomes, each of which is likely to be accompanied by financial stress, and poor health and worse health outcomes has been established [8], [9]. Similarly, an association between financial barriers to healthcare services and worse health outcomes has also been demonstrated [10]. However, while there is likely overlap between financial stress and insurance status, income, and barriers to care, financial stress is the perception by patients of the difficulty affording care and may not be accurately quantified by these other markers of access to care. It may also be independently associated with health and health care outcomes, perhaps through health behaviors and psychological stress. Some individuals may be under severe financial stress, despite having health insurance, a steady income, and few barriers to care. Alternatively, other individuals may not have health insurance or a steady income and face many barriers to care, but not experience financial stress.

Our objective was to examine the association between self-reported financial stress and long-term outcomes while accounting for health insurance coverage and barriers to healthcare services and medications. We studied recovery from acute myocardial infarction (AMI), a common, costly, and often unexpected, acute medical event, such that there is limited financial planning in anticipation of an AMI. Utilizing data from a prospective, multicenter study of patients hospitalized with AMI, we compared the association between self-reported financial stress and patients’ health status, readmission and mortality following hospitalization.

## Materials and Methods

### Sample and Study Design

We utilized data from all patients enrolled in the Prospective Registry Evaluating Myocardial Infarction: Event and Recovery (PREMIER) study, a prospective registry of patients hospitalized with myocardial infarction [11]. Patients with a suspected AMI by positive troponin or creatine kinase-MB fraction were screened for eligibility at 19 participating hospitals in the United States between January 2003 and June 2004 (n = 10,911). Institutional Review Board approval was obtained at each of the 19 participating hospitals. Informed consent was obtained prior to enrollment. Patients were enrolled if they were greater than 18 years of age, presented directly to an enrolling institution or were transferred within 24 hours of the onset of symptoms, had supporting evidence of AMI (ischemic signs and symptoms ST segment changes), and consented. Patients with an elevated troponin or CK-MB secondary to percutaneous coronary intervention and those admitted from penal facilities were not enrolled. Enrolled patients underwent chart abstraction, a baseline interview within 24 to 72 hours of admission, and a 12-month follow-up interview to collect socio-demographic, clinical and treatment data (n = 2,498). For the purposes of our analysis patients were excluded if their disposition was unknown (n = 7), if they left the hospital against medical advice (n = 15), expired in the hospital (n = 17) or if they were discharged to hospice (n = 4), leaving a potential cohort of 2,455 patients.

### Financial Stress

Financial stress was assessed during the baseline interview. While financial stress has been assessed in prior research, there are no commonly accepted measures of self-reported financial stress. Previously published studies have used the following questions: “Have you had problems paying bills or making ends meet in the past year?” [6], “How much do you worry about finances (e.g., money shortage)?” [7], “About money matters, would you say your family has been worse off, the same as, or better off than most other families you know?” [5].

We examined general financial stress using a single question in the baseline interview, which asked, “In general, how do your finances usually work out at the end of the month? Do you find you usually end up with…”, followed by these response choices: “some money left”; “just enough to make ends meet”; and “not enough to make ends meet”. We categorized those individuals who reported some money left as “no stress”, those with just enough to make ends meet as “low stress”, and those with not enough to make ends meet as “high stress”. Among the 2,455 potential patients in our study, 2,344 (95.5%) provided information regarding their general financial stress.

### Main Outcome Measures

We used four measures to examine outcomes among the patients hospitalized for AMI: general health status, disease-specific health status, all-cause readmission, and all-cause mortality. General health status was measured at 1 year after hospitalization using the Short Form-12 (SF-12), a validated instrument assessing general health employing a general physical health scale (PCS) and a mental health (MCS) scale. [12] Both the PCS and MCS are normalized to a mean score of 50 with a standard deviation of 10, where a higher score indicates better health [12].

Disease-specific health status was assessed at 1 year after hospitalization using the Seattle Angina Questionnaire (SAQ), a validated 19-item instrument assessing patients’ perspectives of the impact of ischemic disease [13]. In this study, we assessed two specific domains of the SAQ – angina frequency (SAQ AF) and quality of life (SAQ QoL). Based on patients’ symptoms in the previous 4 weeks, these domains are scored from 0 to 100 where a higher score signifies less angina and better quality of life [13]. A SAQ AF score of 100 reflects no angina and scores less than 100 indicate the presence of angina over the preceding month (0–30 = daily angina, 31–60 = weekly angina and 61–99 = monthly angina).

We also determined readmission to any hospital for any cause within 1 year of hospitalization and mortality from any cause within 4 years of hospitalization. Readmission was assessed through a phone interview 12 months after index hospitalization. Mortality was assessed by cross-referencing patients’ Social Security numbers with the Social Security Master Death File [14]. We used 4 year mortality rates, despite all other outcomes being measured at 1 year, because that was the longest period of follow-up available for analysis.

### Other Variables of Interest

Additional information was collected on all participants, including socio-demographic characteristics, access and barriers to care, clinical characteristics, and other measures of the quality of care participants received within the hospital. Socio-demographic characteristics included age, sex, race, household income, education level, employment status, marital status, and whether the patient lived with others.

Characteristics of access and barriers to care included health insurance coverage, whether the patient had a primary care provider, and self-reported financial barriers to health care. Financial barriers to health care were defined through the baseline interview by two questions. First, “In the past year, have you avoided obtaining health care services because of cost?”, with answer choices of “yes” or “no”. Second, “In the past year, how often have you not taken medication that your doctor prescribed because of cost?”, with answer choices based on a 5-point Likert scale ranging from “never” to “always”. Patients were defined as having experienced financial barriers to health care if they stated they had avoided health care services due to cost or if they stated that they had “occasionally,” “often,” or “always” avoided taking medication due to cost [10].

Clinical characteristics included the type of AMI (with or without ST elevation), ejection fraction less than 40 percent, presence of another acute non-cardiac condition [15], smoking status, body mass index, chronic renal failure, chronic lung disease, diabetes mellitus, hypertension, prior cerebrovacular accident, congestive heart failure, peripheral artery disease, and prior AMI, coronary artery bypass graft or percutaneous coronary intervention.

Measures of quality of care included whether the patient received primary reperfusion (fibrinolytic therapy or primary percutaneous coronary intervention) for ST elevation AMI, the number of performance measures received (maximum eight) and percent of eligible quality indicators received. Eligibility was determined by the prospective abstraction of contraindications for each performance measure. These eight quality indicators included: whether or not the patient received aspirin on arrival, aspirin at discharge, Angiotensin Converting Enzyme (ACE)-inhibitor or Angiotensin II Receptor Blocker (ARB) for left ventricular systolic dysfunction (LVSD) at discharge, smoking cessation instructions, beta blocker at arrival and discharge, and reperfusion for eligible patients [16], [17].

### Statistical Analysis

Baseline socio-demographic characteristics, access and barriers to care, clinical characteristics, and other measures of the quality of care were compared among participants with reported financial stress levels of no, low and high stress using Chi-square or Fisher exact tests for categorical variables and analysis of variance for continuous variables. Highly skewed values (blood pressure, white blood cell count, hemoglobin) were summarized using median and inter-quartile range and tested with the Wilcoxon rank-sum test. Survival estimates were produced using Kaplan-Meier estimates and tested using the log-rank test across reported financial stress levels.

Regression analyses were used to assess the association between financial stress and each of the four main outcome measures independently. For these models, we always adjusted for the patients’ baseline health status and accounted for the clustering of observations by site of enrollment. For example, in measuring angina frequency at 12 months following an AMI, we adjusted for the patients’ baseline angina frequency before their AMI admission. This adjustment produces a statistically equivalent model to one that assesses the change in patients’ health status and avoids the potential bias that those with higher levels of financial stress had worse health status at the time of the admission AMI that in turn accounts for worse health status 1 year later. General health, as measured by SF-12 PCS and SF-12 MCS, and disease-specific quality of life, measured by SAQ QoL, were modeled using within-site hierarchical linear regression. Due to its left skewed distribution, SAQ AF was dichotomized into any angina symptoms (SAQ AF <100) and no angina (SAQ AF = 100) and modeled using a within-site hierarchical modified Poisson regression [18]. All-cause readmission and mortality risk was modeled using proportional hazard regression that accounted for the clustering of observations by site of enrollment.

To assess the independent association of financial stress and outcomes, multivariable models were built, first partially adjusting only for access and barriers to care, and then fully adjusting for socio-demographic, access and barriers to care, clinical, and quality of care variables (see **Tables S1, S2, S3, S4, S5, and S6**). Variables were considered as candidates for inclusion in the model if they differed significantly across financial stress levels, were not highly correlated, and were sufficiently common (≥20). However, because results from the analyses in each step of adjustment were broadly consistent, we only reported results from the unadjusted and fully-adjusted analyses.

Missing information for one or more covariates was minimal; only 6% were missing more than one value. The highest missing rate for any individual variable was 4.5% for baseline SF-12 score. Missing covariate data were assumed to be missing at random (i.e., non-informatively missing given the available observed data) and were imputed in IVEWARE using a single imputation dataset allowing incorporation of all patients into multivariable models [19]. The imputation model consisted of all variables used in the multivariable model in addition to other variables providing information for the imputation (e.g. follow-up scores to impute baseline scores).

Patients could be missing health status or readmission outcome data due to death (n = 199), being too ill (n = 49) or refusing to participate in the 12-month interview (n = 29), or loss to follow-up (n = 261). We evaluated potential bias from missing 12-month outcome data due to patients who were lost to follow-up or refused the 12-month interview. For the overall sample including these patients (but excluding patients who were deceased or too ill to be interview at 12-months follow-up), we calculated a propensity score of having a missing 12-month interview using logistic regression. The propensity score was the probability of a person with given characteristics having a missing 12-month interview. The reciprocal of this score was then used as a weight in the analyses, resulting in higher weight for those patients with similar characteristics as those without follow-up [20]. Models including propensity scores produced similar results. All analyses were performed using SAS version 9.2 (SAS Institute Inc, Cary, NC) and R version 2.11.1 (R Foundation for Statistical Computing, Vienna, Austria).

## Results

### Baseline Characteristics

In our sample of patients admitted for AMI, 1241 (52.9%) reported no financial stress, 735 (31.4%) reported low financial stress, and 368 (15.7%) reported high financial stress (**Table 1**). High self-reported financial stress was associated with several sociodemographic and clinical characteristics, including younger age, lack of employment, lack of insurance, financial barriers to health care, tobacco use and a history of diabetes mellitus, hypertension, and depression.

**Table 1:** Baseline Characteristics of Patients Admitted for Acute Myocardial Infarction, Stratified by Patient Reporting of Financial Stress.

	Financial Stress			
	No Stress (n = 1241)	Low Stress (n = 735)	High Stress (n = 368)	P-Value
**Demographics**				
Age, mean (SD), yrs	62.4 (12.8)	60.3 (13.0)	56.2 (12.1)	<0.001
Male, No. (%)	907 (73.1)	462 (62.9)	213 (57.9)	<0.001
Race/Ethnicity, No. (%)				
White/Caucasian	1046 (84.7)	500 (68.3)	186 (50.8)	
Black/African American	140 (11.3)	197 (26.9)	161 (44.0)	<0.001
Other	49 (4.0)	35 (4.8)	19 (5.2)	
Marital Status, No. (%)				
Married/Common Law	856 (69.6)	396 (54.2)	146 (40.1)	
Widowed	135 (11.0)	115 (15.8)	53 (14.6)	<0.001
Divorced/Separated	158 (12.8)	149 (20.4)	94 (25.8)	
Single/Other	81 (6.6)	70 (9.6)	71 (19.5)	
Live Alone, No. (%)	238 (19.4)	176 (24.3)	93 (26.0)	0.006
Less than High School Education, No. (%)	529 (43.2)	441 (60.5)	234 (64.6)	<0.001
Currently Working for Pay, No. (%)				
Full-time	526 (42.7)	239 (32.7)	76 (20.7)	
Part-time	100 (8.1)	55 (7.5)	36 (9.8)	<0.001
Not currently working for pay	606 (49.2)	437 (59.8)	256 (69.6)	
Household Income, No. (%)				
<$10,000	51 (5.8)	122 (22.1)	128 (47.8)	
$10,000–$29,999	182 (20.6)	228 (41.3)	92 (34.3)	
$30,000–$49,999	222 (25.2)	105 (19.0)	33 (12.3)	<0.001
$50,000–$69,999	143 (16.2)	47 (8.5)	9 (3.4)	
> = $70,000	284 (32.2)	50 (9.1)	6 (2.2)	
**Healthcare Coverage and Utilization**				
Insurance Payor, No. (%)				
Commercial/PPO	627 (52.7)	239 (33.7)	65 (18.5)	
HMO	163 (13.7)	92 (13.0)	28 (8.0)	
Medicare	246 (20.7)	206 (29.0)	94 (26.8)	<0.001
Medicaid	21 (1.8)	54 (7.6)	52 (14.8)	
Other	52 (4.4)	22 (3.1)	25 (7.1)	
None/Self-pay	81 (6.8)	97 (13.7)	87 (24.8)	
Not Taken Medication due to Cost, No. (%)				
Always	6 (0.5)	11 (1.5)	26 (7.1)	
Frequently	8 (0.7)	35 (4.8)	53 (14.6)	
Occasionally	34 (2.8)	66 (9.0)	49 (13.5)	<0.001
Rarely	42 (3.4)	52 (7.1)	31 (8.5)	
Never	1139 (92.7)	566 (77.5)	205 (56.3)	
Avoided Getting Health Care due to Cost, No. (%)	82 (6.7)	170 (23.4)	165 (45.7)	<0.001
Has a Primary Doctor or Care Provider, No. (%)	1058 (85.3)	593 (81.1)	267 (72.6)	<0.001
**Non-Cardiac History**				
Smoked within Last 30 Days, No. (%)	345 (27.9)	257 (35.0)	190 (51.8)	<0.001
Obese (BMI >30), No. (%)	437 (36.4)	301 (43.1)	135 (40.3)	0.01
Chronic Renal Failure, No. (%)	94 (7.6)	88 (12.0)	48 (13.0)	<0.001
Diabetes Mellitus, No. (%)	297 (23.9)	239 (32.5)	134 (36.4)	<0.001
Hypercholesterolemia, No. (%)	625 (50.4)	359 (48.8)	172 (46.7)	0.45
Hypertension, No. (%)	740 (59.6)	499 (67.9)	251 (68.2)	<0.001
Current Taking Medication or In Counseling for Depression, No. (%)	131 (10.6)	85 (11.7)	75 (20.4)	<0.001
**Cardiac History**				
Family History of CAD, No. (%)	418 (33.7)	262 (35.6)	129 (35.1)	0.66
Congestive Heart Failure, No. (%)	122 (9.8)	85 (11.6)	61 (16.6)	0.002
Prior Angina, No. (%)	200 (16.1)	127 (17.3)	75 (20.4)	0.16
Prior CABG Surgery, No. (%)	166 (13.4)	96 (13.1)	44 (12.0)	0.78
Prior PCI, No. (%)	218 (17.6)	127 (17.3)	71 (19.3)	0.69
Prior AMI, No. (%)	243 (19.6)	160 (21.8)	96 (26.1)	0.03
**Acute Presentation**				
Other Acute Non-Cardiac Condition at Presentation, No. (%)	41 (3.3)	37 (5.1)	25 (7.0)	0.008
Final AMI Study Diagnosis, No. (%)				
STEMI	577 (46.5)	316 (43.0)	134 (36.4)	0.002
NSTEMI	664 (53.5)	419 (57.0)	234 (63.6)	
Anterior or Lateral AMI, No. (%)	433 (34.9)	251 (34.1)	119 (32.3)	0.661
Left Ventricular Systolic Function <40%, No. (%)	330 (26.7)	175 (23.8)	98 (26.7)	0.35
Acute Systolic Blood Pressure, median (IQR)	136.0	138.0	132.0	0.09
	(118.0, 158.0)	(119.0, 159.5)	(118.0, 156.0)	
Tachycardia (HR >100), No. (%)	1105 (89.0)	655 (89.1)	328 (89.1)	0.12
WBC Count, median (IQR)	10.0	10.0	9.0	0.06
	(8.0, 12.0)	(8.0, 13.0)	(7.0, 11.0)	
Acute Hemoglobin (g/dL), median (IQR)	13.9	13.4	13.1	<0.001
	(12.2, 15.0)	(12.0, 14.9)	(11.4, 14.9)	
**Inpatient Care**				
Received Fibrinolytic, No. (%)	191 (15.4)	94 (12.8)	35 (9.5)	0.011
Received Primary PCI, No. (%)	632 (50.9)	323 (43.9)	122 (33.2)	<0.001
Received Coronary Angiography (Catheterization, PCI, CABG Surgery), No. (%)	1123 (90.5)	633 (86.1)	296 (80.4)	<0.001
Received Revascularization (PCI, CABG Surgery, Fibrinolytic), No. (%)	956 (77.0)	524 (71.3)	222 (60.3)	<0.001
Received Anti-Platelet Within 24Hours, No. (%)	756 (60.9)	389 (52.9)	169 (45.9)	<0.001
Received Anti-Thrombin, No. (%)	1066 (85.9)	627 (85.3)	312 (84.8)	0.85
Using Aspirin at Admission, No. (%)	473 (38.1)	268 (36.5)	144 (39.1)	0.64
Using Beta Blocker at Admission, No. (%)	377 (30.4)	257 (35.0)	122 (33.2)	0.10
Received Aspirin at Discharge, No. (%)	1162 (93.6)	670 (91.2)	325 (88.3)	0.002
Received Beta Blocker at Discharge, No. (%)	1110 (89.4)	641 (87.2)	303 (82.3)	0.001
Received ACE-Inhibitor or ARB upon Discharge, No. (%)	909 (73.2)	529 (72.0)	274 (74.5)	0.66
Received Instructions for Cardiac Rehabilitation, No. (%)	664 (53.5)	339 (46.1)	128 (34.8)	<0.001
**Quality of Care Measures**				
Number of Eligible Indicators Received, (SD)	4.6 (1.4)	4.5 (1.4)	4.3 (1.4)	<0.001
Percent of Eligible Indicators Received, (SD)	88.7 (16.1)	86.8 (17.8)	85.1 (19.0)	<0.001

**Note:** SD = Standard Deviation; BMI = Body Mass Index; CAD = Coronary Artery Disease; CABG = Coronary Artery Bypass Graft; PCI = Percutaneous Coronary Intervention; AMI = Acute Myocardial Infarction; STEMI = ST-segment Elevation Myocardial Infarction; NSTEMI = Non-ST-segment Elevation Myocardial Infarction; HR = Heart Rate; IQR = Inter-Quartile Range; ACE = Angiotensin Converting Enzyme; ARB = Angiotensin II Receptor Blocker.

At baseline, patients who reported higher financial stress at the time of admission were in worse physical health (mean SF-12 PCS [Standard Deviation (SD)] of 45.4 [11.5], 40.8 [12.7], 37.7 [13.4] among no, low, and high financial stress patients, respectively; p<0.001) and worse mental health (mean SF-12 MCS [SD] of 51.6 [10.6], 49.1 [11.6], 43.8 [13.1] among no, low, and high financial stress patients, respectively; p<0.001; **Table 2**). In addition, patients who reported higher financial stress were more likely to have experienced a diminished quality of life due to their cardiac symptoms (mean SAQ-QoL [SD] of 65.8 [22.1], 60.1 [23.2], 53.9 [26.9] among no, low, and high financial stress patients, respectively; p<0.001) and were more likely to have experienced angina prior to presentation (48.9%, 55.2%, 66.0% among no, low, and high financial stress patients, respectively; p<0.001).

**Table 2:** Health Status at Baseline and at 1 Year of Patients Admitted for Acute Myocardial Infarction, Stratified by Patient Reporting of Financial Stress.

	Financial Stress			
	No Stress (n = 1241)	Low Stress (n = 735)	High Stress (n = 368)	P-Value
**Short Form-12 Physical Component Score, mean (SD)**				
Upon admission	45.4 (11.5)	40.8 (12.7)	37.7 (13.4)	<0.001
1 year	46.9 (11.0)	43.1 (11.6)	38.7 (12.5)	<0.001
**Short Form-12 Mental Component Score, mean (SD)**				
Upon admission	51.6 (10.6)	49.1 (11.6)	43.8 (13.1)	<0.001
1 year	54.6 (8.2)	52.9 (9.7)	48.7 (11.5)	<0.001
**Seattle Angina Questionnaire Quality of Life Score, mean (SD)**				
Upon admission	65.8 (22.1)	60.1 (23.2)	53.9 (26.9)	<0.001
1 year	87.5 (15.2)	84.4 (17.4)	75.0 (24.5)	<0.001
**Seattle Angina Questionnaire Angina Prevalence, No. (%)**				
Upon admission	607 (48.9)	406 (55.2)	243 (66.0)	<0.001
1 year	155 (14.7)	120 (21.3)	92 (36.2)	<0.001

**Note:** SD = Standard Deviation.

### Unadjusted Main Outcomes after AMI by Financial Stress

In unadjusted analyses, one year after admission, general and mental health improved across all groups, but patients reporting higher financial stress remained in worse general health (mean SF-12 PCS [SD] of 46.9 [11.0], 43.1 [11.6], 38.7 [12.5] among no, low and high financial stress patients, respectively; p<0.001) and worse mental health (mean SF-12 MCS [SD] of 54.6 [8.2], 52.9 [9.7], 48.7 [11.5] among no, low and high financial stress patients, respectively; p<0.001). One year after admission, across all groups, fewer patients reported experiencing angina and patients reported less impaired quality of life due to cardiac symptoms. Nevertheless, patients who reported higher financial stress remained more likely to have experienced angina in the prior four weeks (14.7%, 21.3%, 36.2% among no, low and high financial stress patients, respectively; p<0.001) and were more likely to have worse quality of life due to their cardiac symptoms (mean SAQ QoL [SD] of 87.5 [15.2], 84.4 [17.4], 75.0 [24.5] among no, low and high financial stress patients, respectively; p<0.001).

**Figure 1:**
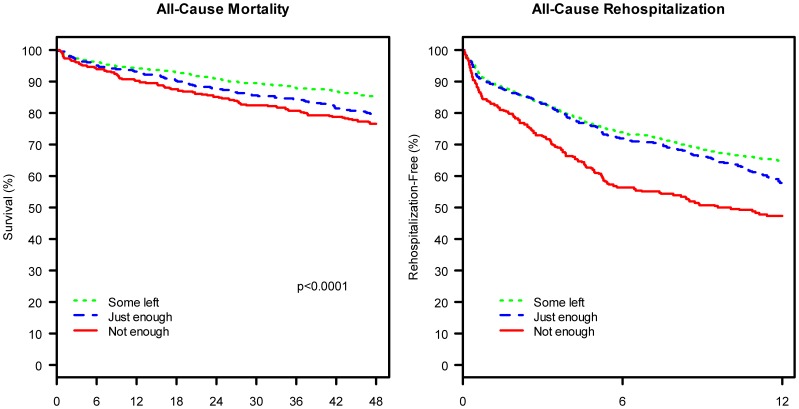
Kaplan-Meier Curves of All-Cause Mortality at 4-Years and All-Cause Rehospitalization at 1-Year among Patients Admitted for Acute Myocardial Infarction, Stratified by Financial Stress. Note: Kaplan-Meier Curves are Unadjusted.

One year after admission, patients who had reported higher financial stress at baseline were more likely to have been readmitted for any cause (35.2%, 42.4%, 52.8% among no, low and high financial stress patients, respectively; p<0.001; **Figure 1**). The hazard ratio of readmission one year after admission was 1.15 (95% CI: 0.98, 1.35) for patients who reported low financial stress and 1.62 (95%CI: 1.33, 1.97) for patients who reported high financial stress when compared with patients who reported no financial stress. Four years after admission, patients who had reported higher financial stress at baseline were at greater risk of death due to any cause (14.8%, 20.7%, 23.5% for no, low and high stress, respectively; p<0.001). The hazard ratio of mortality four years after admission was 1.25 (95% CI: 0.99, 1.56) for patients who reported low financial stress and 1.25 (95%CI: 0.95, 1.65) for patients who reported high financial stress when compared with patients who reported no financial stress.

### Adjusted Main Outcomes after AMI by Financial Stress

**Figure 2:**
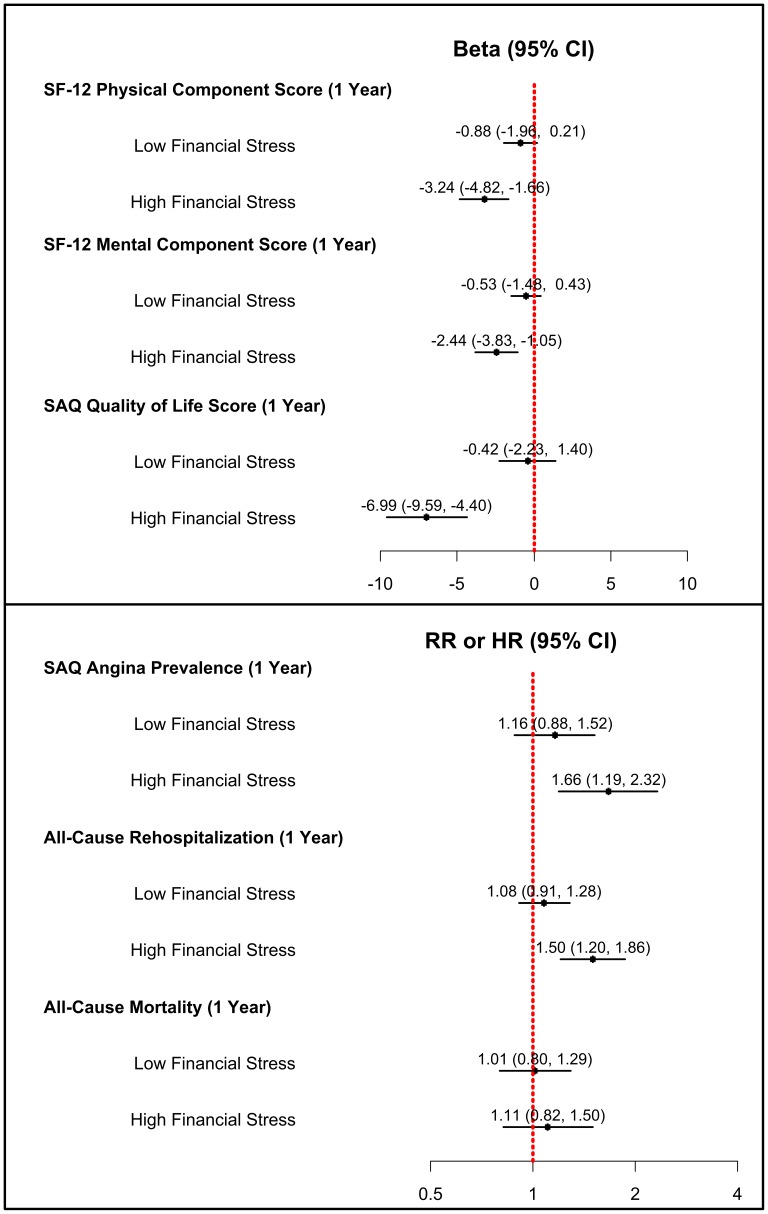
Multivariable Adjusted Health Outcomes at 1-Year and 4-Yearsamong Patients Admitted for Acute Myocardial Infarction, Stratified by Financial Stress. Note: SAQ = Seattle Angina Questionnaire; SF = Short Form. Analyses account for socio-demographic characteristics, access and barriers to care, clinical characteristics, and other measures of quality of care.

In multivariable analysis comparing patients who reported low financial stress with those who reported no financial stress, we found no significant differences in post-AMI outcomes after accounting for socio-demographic, access and barriers to care, clinical, and quality of care variables. Specifically, there was no association between low financial stress and physical health, mental health, angina, quality of life, readmission, or mortality (**Figure 2**).

In contrast, when comparing patients who reported high financial stress with those who reported no financial stress, we found significant associations between stress and outcomes, even after multivariable adjustment. After one year, patients who had reported high financial stress were more likely to be in worse physical health (mean SF-12 PCS difference: −3.24, 95% Confidence Interval [CI]: −4.82, −1.66), more likely to be in worse mental health (mean SF-12 MCS difference: −2.44, 95% CI: −3.83, −1.05), more likely to experience a diminished quality of life due to cardiac symptoms (mean SAQ QoL score difference: −6.99, 95% CI: −9.59, −4.40), and more likely to experience angina (Relative Risk [RR] = 1.66, 95%CI: 1.19, 2.32). In addition, patients reporting high financial stress were at greater risk of all-cause readmission within one year (Hazard Ratio [HR] = 1.50, 95%CI: 1.20, 1.86), although there was no significant association between high financial stress and risk of mortality at 4 years (HR = 1.12, 95%CI: 0.83, 1.53).

## Discussion

In our prospective, multicenter study of patients hospitalized with AMI in 2003 and 2004, nearly half of patients reported some levels of financial stress on admission, and nearly a third of these patients reporting financial stress reported high levels of stress. Today, in an economy burdened by several years of limited economic growth and high unemployment rates, many more patients are likely to be experiencing financial stress. This high financial stress has substantial clinical implications. Not only did patients experiencing high stress have worse clinical symptoms at baseline, but even after accounting for these worse clinical symptoms, patients reporting the highest levels of stress experienced worse outcomes while recovering from AMI. One year after admission they were in worse general health, experienced more cardiovascular symptoms and were more likely to be readmitted to the hospital, although their risk of death at four years was similar to those without financial stress. Moreover, while the general health differences between patients reporting severe financial stress and those reporting no financial stress were modest, not major, differences in physical and mental function, the difference in SAQ scores was equal, or greater, in magnitude than the benefits of percutaneous coronary intervention over optimal medical therapy in the COURAGE trial [21], [22].

As individuals experience high levels of financial stress and have difficulty making ends meet, we would expect that these financial difficulties would translate into barriers to needed and effective health care, such as not taking medicine or avoiding health care services because of costs. Our analysis offers a unique examination of financial stress because we were able to account for income as well as access to care and barriers to care, factors which have been shown to be associated with adverse outcomes [8], [9], [10], in order to better isolate the risk associated with patients’ perception of their financial stress upon admission for AMI. In our study, three-quarters of individuals reporting high levels of financial stress had health insurance and half reported no barriers to care. Even after accounting for access and barriers to care, as well as for patient socio-demographic, clinical, and quality of care variables that are associated with outcomes after AMI, we found that patients reporting the highest levels of financial stress experienced worse outcomes.

Lacking access to health care has serious negative health consequences [8]. A major goal of the recently enacted health care reform legislation was to increase access to care by increasing the number of insured Americans, presumably in an effort to mitigate these adverse health consequences. However, while access to care is critical, attention also needs to be paid to the independent risk conferred by financial stress on health outcomes. Other research has similarly advanced the field in this area, demonstrating that access alone did not ensure appropriate receipt of medical care, but that medical debt remained an independent negative predictor of missing care [23]. As we go forward, these issues that warrant future study and deserve clinical consideration; perhaps patients admitted for acute care should be screened for financial stress in order to identify patient at greater risk for adverse health outcomes after hospitalization.

Our findings should be interpreted in the context of several potential limitations. Although our objective was to determine the association of baseline self-reported financial stress with long-term post-AMI outcomes, we only assessed financial stress at baseline interviews. Patients’ perceptions of their financial situation may have either improved or worsened after their AMI, which would bias our results to the null because of misclassification. In fact, it is quite likely that many patients experienced increased financial stress after admission for AMI, as they may have become unable to work and care for themselves in the same way that they had prior to the event. Second, our findings were observed in a prospective multicenter study performed across many geographic regions that included both academic and nonacademic institutions. However, the results of this study still may not be generalized to the entire population in the United States, particularly to rural populations. Third, the evaluation of financial stress relied on self-reporting, which provides information about the patients’ perspectives. The responses had strong prognostic importance, but we are unable to determine the mechanism by which higher levels of perceived financial stress impact post-AMI outcomes. Finally, we used one measure of financial stress, although prior research has also used others [5]–[7]. This question has specific qualities which we believe makes it a very good measure of financial stress. The specific wording minimizes recall bias and maximizes accuracy of the exposure in this retrospective analysis. Specifying “at the end of the month” ties the respondent a concrete time frame. Additionally, the question evokes a vivid and meaningful response – for the high stress group, the inability to make ends meet.

In conclusion, financial stress is common and is an important risk factor for adverse outcomes post-AMI, independent of access and barriers to care, as well as of other patient socio-demographic, clinical, and quality of care characteristics. Individuals who are unable to make ends meet are vulnerable as high financial stress appears to contribute to poor general health, increased cardiovascular symptoms and increased risk of readmission after admission for AMI. Investigation is needed to further elucidate not only the mechanism by which financial stress may affect outcomes, but also potential interventions.

## Supporting Information

Table S1 **Parameter estimates from unadjusted, partially adjusted, and fully adjusted multivariable models for general health at 1-year as measured using the short form-12 physical component score among patients admitted for acute myocardial infarction, comparing individuals reporting high or low financial stress to individuals reporting no financial stress.**
(DOC)

Table S2 **Parameter estimates from unadjusted, partially adjusted, and fully adjusted multivariable models for mental health at 1-year as measured using the short form-12 mental component score among patients admitted for acute myocardial infarction, comparing individuals reporting high or low financial stress to individuals reporting no financial stress.**
(DOC)

Table S3 **Parameter estimates from unadjusted, partially adjusted, and fully adjusted multivariable models for quality of life at 1-year as measured using the Seattle Angina Questionnaire among patients admitted for acute myocardial infarction, comparing individuals reporting high or low financial stress to individuals reporting no financial stress.**
(DOC)

Table S4 **Risk ratios from unadjusted, partially adjusted, and fully adjusted multivariable models for angina prevalence at 1-year as measured using the Seattle Angina Questionnaire among patients admitted for acute myocardial infarction, comparing individuals reporting high or low financial stress to individuals reporting no financial stress.**
(DOC)

Table S5 **Hazard ratios from unadjusted, partially adjusted, and fully adjusted multivariable models for all-cause readmission at 1-year among patients admitted for acute myocardial infarction, comparing individuals reporting high or low financial stress to individuals reporting no financial stress.**
(DOC)

Table S6 **Hazard ratios from unadjusted, partially adjusted, and fully adjusted multivariable models for all-cause mortality at 4-years among patients admitted for acute myocardial infarction, comparing individuals reporting high or low financial stress to individuals reporting no financial stress.**
(DOC)
