# Supplementary material for: Financial Stress and Outcomes after Acute Myocardial Infarction
Source: PLoS One. 2012 Oct 24;7(10):e47420. doi: 10.1371/journal.pone.0047420 (PMC3480393; doi:10.1371/journal.pone.0047420)
Supplement: Table S5 — Hazard ratios from unadjusted, partially adjusted, and fully adjusted multivariable models for all-cause readmission at 1-year among patients admitted for acute myocardial infarction, comparing individuals reporting high or low financial stress to individuals reporting no financial stress. (DOC) [file pone.0047420.s005.doc]

**Table S5: Hazard ratios from unadjusted, partially adjusted, and fully adjusted multivariable models for all-cause readmission at 1-year among patients admitted for acute myocardial infarction, comparing individuals reporting high or low financial stress to individuals reporting no financial stress. All models account for the clustering of observations by site of enrollment. Partially adjusted multivariable models account only for access and barriers to care; fully adjusted multivariable models account for socio-demographic characteristics, access and barriers to care, clinical characteristics, and other measures of quality of care.**

|  | **Parameter Estimate (95% Confidence Interval)** | | |
| --- | --- | --- | --- |
|  | **Unadjusted** | **Partially Adjusted Model** | **Fully Adjusted Model** |
| **Financial Stress** |  |  |  |
| No Financial Stress | Ref | Ref | **Ref** |
| Low Financial Stress | 1.15 (0.98, 1.35) | 1.12 (0.95, 1.33) | 1.08 (0.91, 1.28) |
| High Financial Stress | 1.62 (1.33, 1.97) | 1.56 (1.27, 1.93) | 1.50 (1.20, 1.86) |
| **Healthcare Access and Barriers** |  |  |  |
| Payer |  |  |  |
| Commercial/PPO | -- | 1.03 (0.83, 1.27) | 1.00 (0.81, 1.24) |
| HMO | -- | 0.81 (0.60, 1.10) | 0.83 (0.61, 1.13) |
| Medicare | -- | 1.09 (0.92, 1.29) | 1.01 (0.81, 1.25) |
| Medicaid | -- | 1.12 (0.86, 1.45) | 1.00 (0.76, 1.33) |
| Other | -- | Ref | Ref |
| None/Self-pay | -- | 0.87 (0.66, 1.15) | 0.90 (0.68, 1.20) |
| Financial Barrier to Care | -- | 1.16 (0.97, 1.38) | 1.11 (0.93, 1.33) |
| Has a Primary Doctor or Care Provider | -- | 1.13 (0.92, 1.38) | 1.01 (0.82, 1.25) |
| **Demographics** |  |  |  |
| Age | -- | -- | 0.99 (0.98, 1.00) |
| Male | -- | -- | 0.85 (0.72, 1.01) |
| Race/Ethnicity |  |  |  |
| White/Caucasian | -- | -- | Ref |
| Black/African American | -- | -- | 0.82 (0.65, 1.03) |
| Other | -- | -- | 0.92 (0.64, 1.32) |
| Marital Status |  |  |  |
| Married/Common Law | -- | -- | Ref |
| Widowed | -- | -- | 0.86 (0.66, 1.12) |
| Divorced/Separated | -- | -- | 0.88 (0.71, 1.11) |
| Single/Other | -- | -- | 0.73 (0.54, 0.98) |
| Live Alone | -- | -- | 1.06 (0.86, 1.30) |
| Less than High School Education | -- | -- | 0.86 (0.74, 0.99) |
| Currently Working for Pay |  |  |  |
| Full-time | -- | -- | 0.77 (0.63, 0.95) |
| Part-time | -- | -- | 0.98 (0.76, 1.27) |
| Not currently working for pay | -- | -- | Ref |
| **Non-Cardiac History** |  |  |  |
| Smoked within Last 30 Days | -- | -- | 0.95 (0.79, 1.13) |
| Obese (BMI > 30) | -- | -- | 1.00 (0.99, 1.01) |
| Chronic Renal Failure | -- | -- | 1.28 (1.00, 1.63) |
| Diabetes | -- | -- | 1.14 (0.97, 1.34) |
| Hypertension | -- | -- | 1.18 (1.00, 1.39) |
| History of CVA | -- | -- | 0.98 (0.73, 1.31) |
| History of Pulmonary Disease | -- | -- | 1.14 (0.92, 1.40) |
| Use of Anti-Depressant Medication on Admission | -- | -- | 1.08 (0.79, 1.48) |
| Anti-Depressant Medication Prescribed at Discharge | -- | -- | 1.04 (0.77, 1.41) |
| **Cardiac History** |  |  |  |
| Prior AMI | -- | -- | 1.26 (1.07, 1.48) |
| Prior Congestive Heart Failure | -- | -- | 1.25 (0.99, 1.57) |
| Prior Peripheral Arterial Disease | -- | -- | 1.41 (1.10, 1.82) |
| **Acute Presentation** |  |  |  |
| Other Acute Non-Cardiac Condition at Presentation | -- | -- | 1.00 (0.71, 1.41) |
| Final AMI Study Diagnosis |  |  |  |
| STEMI | -- | -- | 1.01 (0.83, 1.23) |
| NSTEMI | -- | -- | Ref |
| Primary Reperfusion | -- | -- | 1.07 (0.87, 1.30) |
| Left Ventricular Systolic Function <40% | -- | -- | 1.25 (1.05, 1.50) |
| **Quality of Care Measures** |  |  |  |
| Number of Eligible Indicators Received | -- | -- | 1.04 (0.96, 1.12) |
| Percent of Eligible Indicators Received | -- | -- | 1.00 (1.00, 1.00) |

**Note:** BMI=Body Mass Index; AMI=Acute Myocardial Infarction; STEMI=ST-segment Elevation Myocardial Infarction; NSTEMI=Non-ST-segment Elevation Myocardial Infarction.
